# Extraction, characterization, and utilization of mung bean starch as an edible coating material for papaya fruit shelf‐life enhancement

**DOI:** 10.1002/fsn3.4166

**Published:** 2024-05-19

**Authors:** Madhu Sharma, Aarti Bains, Sanju Bala Dhull, Prince Chawla, Gulden Goksen, Nemat Ali

**Affiliations:** ^1^ Department of Food Technology and Nutrition Lovely Professional University Phagwara Punjab India; ^2^ Department of Microbiology Lovely Professional University Phagwara Punjab India; ^3^ Department of Food Science and Technology Chaudhary Devi Lal University Sirsa Haryana India; ^4^ Department of Food Technology, Vocational School of Technical Sciences at Mersin Tarsus Organized Industrial Zone Tarsus University Mersin Turkey; ^5^ Department of Pharmacology and Toxicology, College of Pharmacy King Saud University Riyadh Saudi Arabia

**Keywords:** coating, papaya fruit, shelf‐life improvement, starch

## Abstract

This research was aimed to investigate the utilization of mung bean starch as an innovative edible coating material to enhance the shelf‐life of cut papaya fruits. The study focused on the extraction process of mung bean starch and its subsequent characterization through various analyses. Particle size (142.3 ± 1.24 nm), zeta potential (−25.52 ± 1.02 mV), morphological images, Fourier transform infrared (FTIR) spectra, and thermal stability (68.36 ± 0.15°C) were assessed to determine the mung bean starch properties. The functional properties, such as bulk density (0.51 ± 0.004 g/cm^3^) and tapped density (0.62 ± 0.010 g/cm^3^), angle of repose (21.61°), swelling power (12.26 ± 0.25%), and minimum gelation concentration (4.01 ± 1.25%), were examined to detect its potential as a coating base material. Subsequently, the prepared mung bean starch coating solution (1%, 2%, 3%, 4%, and 5%) was applied to papaya fruits and the coated fruits' physicochemical characteristics evaluated during storage. These characteristics encompassed color, weight loss, pH shifts, total soluble solids, titratable acidity, vitamin C content, fruit firmness, microbial analysis, and sensory attributes. The results revealed that starch coating on papaya maintained its color, reduced weight loss, preserved vitamin C, and delayed firmness loss, enhancing shelf‐life when compared to control sample. These findings demonstrated the effectiveness of mung bean starch coatings in preserving papaya fruits. The research made a significant contribution to the use of mung bean starch as a potential coating material for improving the shelf‐life of papaya fruits. This finding has great promise for the field of food preservation and quality control.

## Graphical abstract

This research was aimed to investigate the utilization of mung bean starch as an innovative edible coating material to enhance the shelf‐life of cut papaya fruits. The study focused on the extraction process of mung bean starch and its subsequent characterization through various analyses. The research made a significant contribution to the use of mung bean starch as a potential coating material for improving the shelf‐life of papaya fruits.

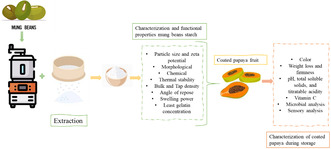

## 1 INTRODUCTION

In recent years, the demand for sustainable and innovative ways to enhance the shelf‐life of minimally processed foods has led researchers to explore the novel methods of preservation (Al‐Tayyar et al., 2020). Among these methods, the utilization of edible coatings on fruits and vegetables has emerged as a promising strategy to extend their shelf‐life while ensuring their quality and safety. Fruits and vegetables, which are high in vitamins, are highly perishable and susceptible to microbial deterioration. Natural antimicrobial coatings have proven to be an excellent alternative for extending the shelf‐life of food. It has been seen that papaya fruit consumption has increased due to its wide‐ranging nutritional and health benefits, including the prevention of fatal diseases such as cancer, arteriosclerosis, and heart problems (Kong et al., 2021). Thus, the primary goal of this research is to develop an edible coating for papaya, which has high nutritional value but suffers significant postharvest losses due to its short shelf‐life. Furthermore, minimal processing of papaya results in higher respiration rates than whole fruit, possibly due to the increased surface area exposed to the atmosphere and the increased metabolism of injured or exposed cells. The preservation of minimally processed fruits is a critical concern, as their rapid deterioration not only results in economic losses but also contributes to food waste and scarcity (Messner et al., 2021). Traditional methods of extending shelf‐life, such as refrigeration and chemical preservatives, have raised environmental and health concerns. Edible coatings, on the other hand, offer a sustainable alternative that aligns with the growing demand for natural, eco‐friendly, and safe food preservation techniques (Roy et al., 2023).

Mung bean (*Vigna radiata*), a leguminous crop, is valued for its nutritional content and diverse applications in the food industries. It contains starch with distinctive properties that make them suitable for innovative applications (Baranwal et al., 2022). Starch, as a biopolymer, is a polysaccharide composed of amylose and amylopectin. Its functional versality, biodegradability, and compatibility with human consumption have driven interest in utilizing starch‐based materials for food‐related applications. It can be used as edible coatings for fruits and vegetables, and these coatings serve as a protective barrier that minimizes water loss, gas exchange, and microbial growth (Nicolau‐Lapeña et al., 2021). The choice of coating material significantly influences its performance and effectiveness.

The primary objective of this study is to extract and characterize mung bean starch for its suitability as an edible coating material. The application of mung bean starch‐based coatings on papaya fruits will be investigated, aiming to enhance their shelf‐life by mitigating the adverse effects of moisture loss, oxidative processes, and microbial activity. The utilization of mung bean starch as an edible coating material holds significant promise for the food industries. The application of such coatings can lead to reduce losses of minimally processed fruits, increase availability, and decrease the use of chemical preservatives. In addition, the biodegradable nature of these coatings aligns with the sustainability goals and addresses concerns related to environmental impact. In conclusion, the investigation into the extraction, characterization, and utilization of mung bean starch as an edible coating material for enhancing papaya cut‐fruit's shelf‐life has been conducted. This study aims to contribute to the development of innovative and sustainable approaches to food preservation.

## 2 MATERIALS AND METHODS

### 2.1 Chemicals and reagents

Chemicals used in the study including sodium metabisulfite (Na_2_S_2_O_5_), sodium hydroxide (NaOH), and glycerol were acquired from Sigma Aldrich Chemicals Pvt. Ltd. (Delhi, India). Nutrient agar and potato dextrose agar (PDA) were purchased from Neogen Chemicals Pvt. Ltd. (Mumbai, Maharashtra, India). All the reagents and chemicals used throughout the research were of analytical grade.

### 2.2 Collection of raw material

The mung bean legume seeds were purchased from the market of Phagwara (Punjab, India) for the starch extraction and the fresh papaya was harvested from the farms of Lovely Professional University for the coating application.

### 2.3 Extraction of starch

The starch from mung bean seeds was extracted according to Chettri et al. (2023). The 0.5 kg of mung bean seeds was immersed in sodium metabisulfite solution (0.5%) and left overnight at 4°C, which was followed by homogenization in a laboratory grinder (Philips HL 7720, Philips, Amsterdam, the Netherlands) and filtration using a sieve (100 μm). The filtrate was combined with water and filtered repeatedly 4–5 times, which was then left at room temperature for 2 h for sedimentation. The upper layer of the residual protein was removed using a spatula and the remaining starch was reslurried and washed again to ensure its purity. Finally, the obtained starch was dried in a hot air oven at 50°C for 15–16 h. After drying, the starch was finely ground and sieve through a 150‐μm mesh screen, which was then packed in polypropylene bags and stored for further research analysis.

### 2.4 Characterization of starch

#### 2.4.1 Particle size and zeta potential

The particle size and surface charge of the mung bean starch were determined at 25°C using dynamic light scattering (Mastersizer 3000, Malvern Instruments Ltd., Malvern, Worcestershire, UK) (Lin et al., 2020; Sharma et., 2023). The sample (1% w/v) was prepared by dispersing 500 mg of mung bean starch powder in 50 mL of ultrapure water. The samples were scanned three times using fresh cuvettes for each sample respectively.

#### 2.4.2 Morphological characteristics

The method was carried out to study the morphological characteristics of starch granules using a field emission scanning electron microscope (FEI Nova NanoSEM 230, FEI Company, USA) (Rahaman et al., 2021). The powdered starch (5 mg) was fixed on the aluminum specimen holder with conductive paste and coated with gold palladium at 20 mA for 2 min. Micrographs were captured at 500× and 10,000× magnifications at a working distance of 8.0 and 8.2 mm, respectively, with an accelerating voltage of 15 kV.

#### 2.4.3 FTIR

The existing functional groups of mung bean seed starch were determined using Bruker Vertex 70 FT‐IR spectrometer (Bruker Optics, USA) (Govindaraju et al., 2021; Patil et al., 2023). The mung bean starch powder (10 mg) was prepared by mixing with 100 mg of potassium bromide (KBr) to create pellet samples. The spectra were recorded at 4000 to 400 cm^−1^ wavenumber range keeping air as the background and resolution set at 4 cm^−1^. OPUS software 7.0 was used for measuring the transmittance.

#### 2.4.4 Thermal stability

The thermal behavior of powdered mung bean seed starch was analyzed using differential scanning calorimetry (DSC) (Q20, TA Instruments, New Castle, NJ, USA) (Li, Hu, et al., 2021; Li, Zhang, et al., 2021). The calibration of the instrument for heat and temperature flow was performed using a combination of indium and zinc. The powdered starch sample (5 mg) was placed into an aluminum DSC pan (PerkinElmer, 0219‐0071) and diluted with water (10 mL) and measurements were recorded at a 10°C/min scan rate from 10 to 450°C while an empty pan was used as a reference.

### 2.5 Functional properties of starch

#### 2.5.1 Bulk and tapped density

The bulk and tapped density of the mung bean starch powder was determined according to the methodology put forth by Balci‐Torun and Ozdemir (2021). Briefly, 5 g of mung bean starch was poured into a 50 mL of measuring cylinder and the weight/volume ratio (kilogram per cubic meter (kg/m^3^)) was calculated to determine the bulk density, while for the tapped density the 5 g of powdered starch sample was poured into a measuring cylinder and then tapped on a hard surface until constant volume was observed. The weight/volume (kg/m^3^) ratio was used to calculate tapped density. The experiments for both bulk and tapped density are carried out in triplicate.

#### 2.5.2 Angle of repose

The angle of repose for the powdered mung bean starch was determined with slight modification (Kenekar et al., 2023). Briefly 100 g of powdered mung bean starch was dropped through the funnel until a cone‐shaped pile formed on the surface. The height (*h*) and radius (*r*) of the formed pile were measured by a pre‐calibrated scale. Following Equation (1) was utilized to calculate the angle of repose.

(1) $$\tan\left(\theta \right)=\left[\left(\frac{\text{height of formed cone}‐\text{shaped pile}}{\text{radius of the formed cone}‐\text{shaped base}}\right)\right]$$

#### 2.5.3 Swelling power

The swelling power of powdered mung bean starch was measured using the method described by (Huang et al., 2018). Briefly, 1 g of starch sample was weighed (W1) and mixed with 25 mL of distilled water. The mixture was heated at a controlled temperature of 80°C for 30 min with continuous stirring. After cooling, the centrifugation was performed at 5000 × *g* for 20 min and the combined weight of supernatant and precipitate (W2) was measured. Following Equation (2) was showed to measure the swelling power of starch:

(2) $$\text{Swelling power}=\left[\left(\frac{W2-W1}{W1}\right)\times 100\right]$$

#### 2.5.4 Least gelatin concentration

The least gelation concentration of powdered mung bean starch was determined using the method developed by Olagunju et al. (2020). Initially, the starch solution of varying concentrations from 2 to 30% (w/v) (at 2% intervals) was prepared and filled in separate test tubes followed by heating at 90°C for 30 min. while stirring. After cooling, the solutions were observed for gel formation, and the least concentration was identified as the least concentration at which the starch sample did not slip when the test tubes were inverted.

### 2.6 Preparation of coating solution and application on treatments

The coating solution of mung bean starch was prepared at the different concentrations of starch (Chettri et al., 2023). Briefly, the different amounts of starch (1%, 2%, 3%, 4%, and 5% w/v) were taken and 100 mL of water was added to each sample, followed by the addition of 3 mL of glacial acetic acid and 1.5 mL of glycerol. Individually, the resulting solution was subjected to homogenization by placing each sample on a magnetic stirrer for 25–30 min at a temperature of 80°C.

The fresh papaya fruits having uniform color were selected and subjected to initial disinfection with sodium hypochlorite solution (0.1%), followed by rinsing with distilled water, peeling using a clean, sharp stainless steel knife, deseeding, and finally cutting the fruit into small equal‐sized pieces (2 × 2 cm). The coating was then applied to fresh‐cut papaya fruit by immersing into the coating solution (at different concentrations) for 3 min, followed by 60 min drying at an ambient temperature. A control group (uncoated samples) was also included. Both coated and uncoated samples were then placed in polyethylene trays (2 cm × 4 cm) and covered to prevent moisture and aroma loss. Subsequently, the physicochemical and microbiological changes in the samples were evaluated during a 12‐day storage period at 4–7°C. Th analysis was conducted at an interval of 3 days.

### 2.7 Physicochemical characteristics of coated papaya

#### 2.7.1 Color measurement

The color of papaya fruit was quantified using the HunterLab Colorimeter (ColorFlex EZ, HunterLab, Murnau, Germany) by following the method of Ma et al. (2021). The L*, a*, and b* values correspond to lightness (100 for white and 0 for black), red–green chromaticity (−80 for green and 80 for red), and yellow–blue chromaticity (−80 for blue and 80 for yellow), respectively. This measurement provides the fresh‐cut fruit color, including its brightness, hue, and saturation. The study utilized the mean values from the three replications executed for each experiment.

#### 2.7.2 Weight loss and firmness

The weight changes of coated and control papaya fruits were determined at regular intervals (0, 3, 6, 9, and 12 days of storage) using a high‐precision laboratory scale (Wensar analytical balance MAB201/301, India), following the methodology outlined by Parven et al. (2020). The weight loss was calculated using the following Equation (3) and expressed as a %.

(3) $$\text{Weight loss}\;\left(\%\right)=\left[\left(\frac{\text{Initial weight of papaya}\;\left(g\right)-\text{final weight of papaya}\;\left(g\right)}{\text{Initial weight of papaya}\;\left(g\right)}\right)\times 100\right]$$

The firmness of coated and uncoated papaya fruits was assessed using a hand‐held penetrometer (M/s QA Supplier USA, Model: FT 327) (Chettri et al., 2023). The probe was inserted into the coated fruit samples to a consistent depth, and firmness was determined by measuring the rupture force.

#### 2.7.3 pH, total soluble solids (TSS), and titratable acidity (TA)

The methodology proposed by de Oliveira Filho et al. (2022) was used to evaluate change in pH of the sample. In brief, 1 g of papaya sample was crushed and mixed with 20 mL of distilled water. The pH was measured using a pH meter (LAQUAtwin‐pH‐22, Horiba Scientific, Japan).

The TSS content of coated and control papaya samples was assessed using a hand refractometer (ERMA Inc., Tokyo, Japan) and the results were presented in °Brix (°B) (Vieira et al., 2020).

For the determination of TA, 1 g of coated and control papaya samples was crushed and mixed with 20 mL of distilled water. The mixture was titrated against 0.1 N alkali solution (NaOH) with phenolphthalein as an indicator and acidity was expressed as percentage (%) relative to citric acid.

#### 2.7.4 Vitamin C analysis

Vitamin C content of coated and control papaya fruits was determined according to Ponder and Hallmann (2020) by titrating with 2,6‐dichlorophenolindophenol (DCPIP). It was expressed in milligrams per 100 grams (mg/100 g).

#### 2.7.5 Microbial analysis

The effect of storage on total plate, yeast, and mold counts was analyzed on the 0th, 3rd, 6th, 9th, and 12th days (Tabassum & Khan, 2020). In brief, a 1 g sample was manually crushed and blended with 20 mL of distilled water using a mortar and pestle, followed by serial dilution. Subsequently, 0.1 mL of the diluted sample was placed onto Petri plates to enumerate total plate, yeast, and mold counts. Incubation periods and temperatures were 37°C for 48 h for total plate count and 25°C for 72 h for yeast and mold counts, respectively.

#### 2.7.6 Sensory analysis

Male and female semi‐trained panelists (*n* = 35), ages 25–45 years, evaluated the sensory assessment of coated and control sliced papaya. The sensory analysis was conducted in a sensory laboratory with a separate sensory analysis booth designed according to the guidelines provided by the International Organization for Standardization EC/LPU/23/215. Papaya samples were presented on dishes marked with random, anonymous codes to reduce the potential for bias. The panelists evaluated attributes, such as texture, flavor, appearance, and overall acceptability, using a 9‐point hedonic scale.

### 2.8 Statistical analysis

The experiments were conducted three times each, and the outcomes are presented as the mean value along with the standard deviation. The data underwent analysis of variance (ANOVA), and Duncan's multiple range tests, available through SPSS statistical software version 17.0 (SPSS, Inc, Chicago, IL, USA), were utilized. A significance level of *p* ≤ .05 was adopted to ascertain statistical significance.

## 3 RESULTS AND DISCUSSION

### 3.1 Characterization of starch

#### 3.1.1 Particle size and zeta potential

**FIGURE 1:**
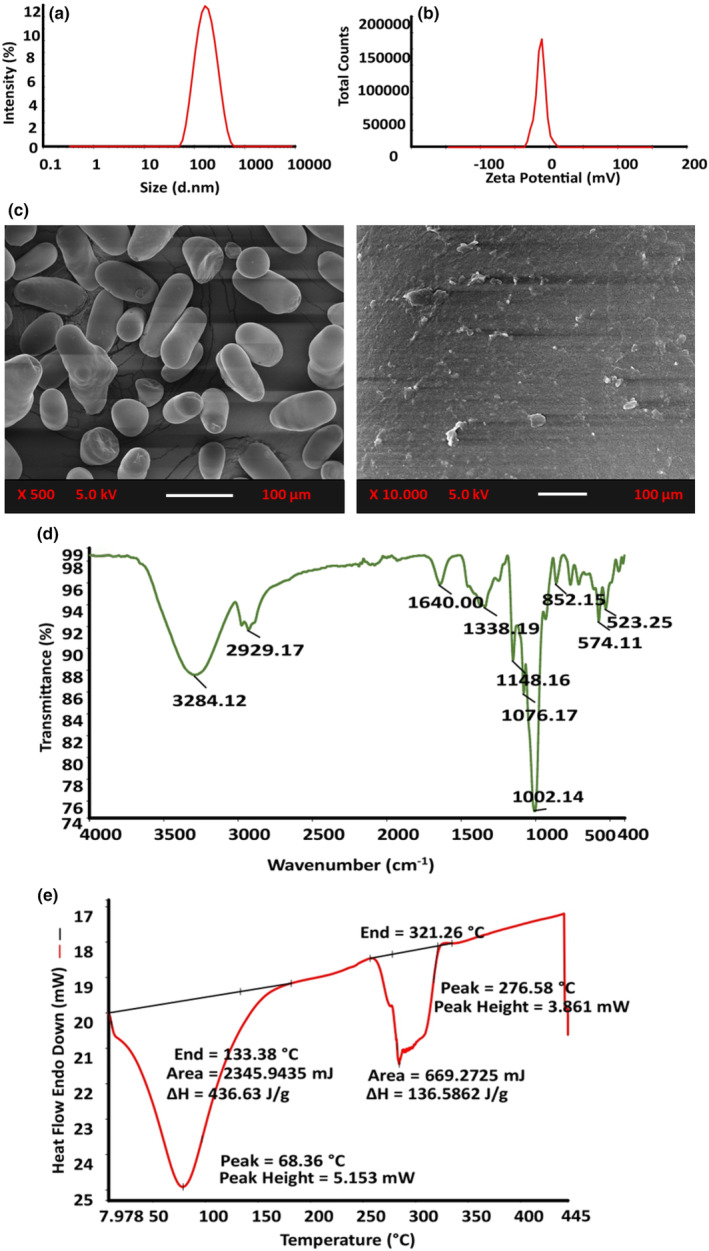
Characterization of mung bean starch using different analytical techniques: (a) particle size distribution, (b) zeta potential, (c) scanning electron microscopy images (500 and 10,000× magnifications), (d) Fourier transform infrared (FTIR) spectrum, and (e) differential scanning calorimetry (DSC) thermograph.

The particle size distribution of the extracted starch was evaluated using dynamic light scattering (DLS) technique, which was found to be 142.3 ± 1.24 nm with polydispersity index of 0.34, as shown in Figure 1a. The particle size could be attributed to various factors, including sources, extraction processes, and the inherent nature of starch molecule. These factors may influence the formation of starch aggregates or clusters, leading to the variability in particle size. The surface charge of the extracted mung bean starch was determined as −25.52 ± 1.02 mV, as presented in Figure 1b. The measured zeta potential indicates the potential for colloidal stability and instability. The higher zeta potential value is indicative of the electrostatic repulsion between particles, resulting in improved colloidal stability and reduced aggregation (Kumari et al., 2022). Similar results have been reported by Purwandari et al. (2023) for jack bean starch. Overall, the consistent particle size and stable dispersion are the key factors for the product quality.

#### 3.1.2 Morphological characteristics

The morphological characteristics of the mung bean starch are indicated in Figure 1c. The image analysis revealed that the particles were elliptical and oval shaped with smooth surface, which might be characteristics of plant originated starch (Martins et al., 2022). There is no damage or rupture of granules observed, which indicates that the extraction process was effective. Furthermore, the absence of impurities underscores the efficiency of the extraction process. Mung bean starch granules' diameter was observed to be 15–25 μm. Similar results have been observed by Yao et al. (2019) for the morphological evaluation of mung bean starch. Overall, these structural attributes have implications for the starch functional behavior in various food applications.

#### 3.1.3 FTIR

The structural attributes of mung bean starch are illustrated in Figure 1d. A distinct peak at 3284.12 cm^−1^ signifies strong O–H stretching vibrations, indicating both intra‐ and intermolecular hydrogen bonding and peak at 2929.17 cm^−1^ correspond to C–H stretching/bending, arising from the sugar methyl group. Additionally, the presence of water molecules absorbed within the starch is indicated by the peak at 1640.00 cm^−1^, depicting bending vibrations. Furthermore, the bands observed at 1338.19 cm^−1^, 1148.16 cm^−1^, 1076.17 ^−1^, and 1002.14 cm^−1^ are attributed, respectively, to CH_2_ bending vibrations, C=O stretching vibrations of C–O–H, and C–O–C groups within glucose sugar (Huong et al., 2021). Notably, vibrations linked to the α‐1,4 glycosidic linkage's C–O stretching are evident around 852.15 cm^−1^, 574.11 cm^−1^, and 523.25 cm^−1^. These findings align with the reported spectral characteristics of mung bean seed starch by (Nadaf et al., 2021), confirming the presence of significant functional groups corresponding to vital starch components. These functional groups offer insights into starch's molecular structure and potential bioactive compounds, holding promise for applications in the food and pharmaceutical sectors (Jindal et al., 2023). Mung bean seed starch shares functional similarities with other plant‐based starches (Vogelsang‐O'Dwyer et al., 2021), potentially serving as agents for thickening, gelling, and encapsulation in various industries.

#### 3.1.4 Thermal stability

The thermal behavior of mung bean seed starch was analyzed by DSC, and the thermogram is presented in Figure 1e, which depicts the heat flow as a function of temperature. A prominent endothermic peak was observed at 68.36 ± 0.15°C, corresponding to the gelatinization process of mung bean starch. This peak indicates the release of energy (436.63 J/g) as the starch granules undergo structural changes due to disruption of intermolecular hydrogen bonds and rearrangement of molecules. Various factors, including amylose content, relative crystallinity, and starch structure, influenced the gelatinization temperature of the mung bean starch. Amylose present in the starch majorly contributes to the amorphous region, which starts softening and hence gelatinized. A broad peak at 276.58°C signifies the melting process with 138.28 J/g enthalpy. This transition point reflects the breakdown of the crystalline structure of starch. The observed thermal behavior of mung bean starch aligns with the study of Li, Hu, et al. (2021) and Li, Zhang, et al. (2021) for yard‐long bean starch. Overall, the gelatinization temperature and melting points are essential parameters that influence the functional properties of starch in various applications.

### 3.2 Functional properties of mung bean starch

The bulk and tapped density of mung starch was found to be 0.51 ± 0.004 g/cm^3^ and 0.62 ± 0.010 g/cm^3^, respectively. These values were primarily influenced by various factors, including particle size, particle arrangements, and moisture content. Comparable results have been found by Nadaf et al. (2021) for the mung bean starch. The minimal disparity exhibited limited interparticle interactions. This enhanced flowability may be due to its uniform particle size distribution and reduced moisture content.

The angle of repose is an indicative factor of interparticle friction or particle cohesion, which was 21.61° for mung bean starch. A lower angle of repose signifies particles that are smooth, rounded, and non‐cohesive, whereas a higher angle of repose corresponds to finer and stickier material. Materials with lower angle of repose values typically exhibit free‐flowing characteristics, often relying on gravitational force for transportation and requiring minimal energy input. Similar results have been reported by Chettri et al. (2023) for mung bean starch.

The swelling power of mung bean starch was found to be 12.26 ± 0.25%, which can vary according to the morphology of starch granules, encompassing factors, such as particle size distribution, surface characteristics, crystallinity, water absorption capability, and amylose content. According to the findings by Rengadu et al. (2020), starches possessing lower amylose content exhibit greater levels of swelling compared to starches characterized by higher amylose content. The results aligned with the findings of Sharma et al. (2021) for the kidney bean starch.

The lowest gelation concentration of the extracted mung bean starch was found to be 4.01 ± 1.25% (w/v). Water absorption by starch leads to the release of amylose and amylopectin within the polymer network, ultimately giving rise to a three‐dimensional (3D) matrix that constitutes the gel. This phenomenon disrupts the starch structure, resulting in the creation of interlinked chains that effectively entrap water, culminating in the formation of a coherent gel framework. The overall resilience of this starch gel structure hinges on the presence of negatively charged groups, including carbonyl, acetyl, and carboxyl groups. Increased concentrations of these groups induce increased electrostatic repulsion forces, thereby hindering the establishment of robust bonds and consequently diminishing the stability of the starch gel network. Our results are similar to the findings of Chettri et al. (2023) who observed the least gelation concentration of starch was 3%.

### 3.3 Physicochemical characteristics of coated papaya

#### 3.3.1 Color

As shown in Table 1, the effect of starch coating on the color of papaya cut‐fruit was evaluated during 12 days of storage at 4–7°C. Initially at 0 day, the coated sample's S1% (50.86 ± 0.37), S2% (50.01 ± 0.11), S3% (49.67 ± 0.65), S4% (48.35 ± 0.43), and S5% (47.99 ± 0.19) were not significantly (*p* > .05) different from the control sample's L* value of (51.14 ± 0.60). Both samples' lightness (L*) values decreased during storage, which suggests that the color darkened. The coated samples, S1% (39.86 ± 0.75), S2% (40.08 ± 0.12), S3% (40.42 ± 0.18), S4% (40.53 ± 0.61), and S5% (40.53 ± 0.61), however, showed significantly higher values (*p* < .05) than the control samples (36.22 ± 0.5) on the 12th day of analysis. The decrease in L* value (lightness) indicates ripeness or senescence of papaya, which was observed to be less in starch‐coated fruit, indicating delayed ripening. This could be due to starch, which acts as an oxygen barrier, protecting carotenoids from oxidation and thus contributing to the lightness of fresh‐cut papaya fruits. The mean a* value of the control sample increased significantly as compared to coated samples over 12 days of storage. The insignificant increase in the coated samples indicates the lower biochemical changes in the fruits, which also delayed ripening and hence increased the shelf‐life of cut papaya fruits. Moreover, the mean b* value of the control sample (14.58 ± 0.13) decreased significantly (*p* < .05) as compared to the coated samples; S1% (15.29 ± 0.45), S2% (16.86 ± 0.46), S3% (17.58 ± 1.16), S4% (18.32 ± 0.26), and S5% (19.09 ± 0.37). The decrease in the b* value of control fruits indicated rapid ripening or senescence of papaya fruit, whereas the coated sample preserved the yellowish color and prevented oxidative damage. Similar results have been reported in the study conducted by Kathiresan and Lasekan (2019) during the color analysis of fresh‐cut papaya fruit. Overall, the color stability of the starch‐coated sample is better, as evidenced by a smaller decrease in L*, a*, and b* values when compared to the control sample.

**TABLE 1:** Color characteristics of starch‐coated papaya cut‐fruits over 12 days of storage at 4°C.^1^

Storage time (days)						
		0	3	6	9	12
L*	S0%	51.14 ± 0.60^aE^	47.67 ± 0.8^eD^	38.83 ± 0.91^aC^	34.00 ± 0.3^aA^	36.22 ± 0.5^aB^
	S1%	50.86 ± 0.37^aD^	47.31 ± 0.59^dC^	39.86 ± 0.62^bA^	42.22 ± 0.21^bB^	39.86 ± 0.75^bA^
	S2%	50.01 ± 0.11^aE^	46.99 ± 0.52^cD^	40.64 ± 0.51^cB^	42.26 ± 0.45^bC^	40.08 ± 0.12^cA^
	S3%	49.67 ± 0.65^aE^	46.34 ± 0.57^cD^	41.17 ± 0.43^dB^	42.31 ± 0.27^bC^	40.42 ± 0.18^cA^
	S4%	48.35 ± 0.43^aD^	46.08 ± 0.62^bC^	42.42 ± 0.51^eB^	42.59 ± 0.18^bB^	40.53 ± 0.61^cA^
	S5%	47.99 ± 0.19^aE^	45.88 ± 0.61^aD^	43.08 ± 0.31^fC^	42.27 ± 0.2^bB^	41.20 ± 0.90^cA^
a*	S0%	17.29 ± 0.7^bA^	23.06 ± 1.1b^eC^	24.14 ± 0.2^bD^	22.25 ± 0.4^aB^	27.25 ± 1.2^bES^
	S1%	17.21 ± 0.4^bA^	22.31 ± 0.54^dB^	23.96 ± 0.26^bD^	22.86 ± 1.21^bC^	25.68 ± 0.6^aE^
	S2%	17.04 ± 0.48^bA^	21.93 ± 0.16^cB^	23.61 ± 0.95^bD^	22.93 ± 1.14^bC^	24.72 ± 0.69^aE^
	S3%	16.91 ± 0.10^bA^	21.68 ± 0.19^cB^	23.45 ± 0.13^bD^	23.12 ± 1.19^bC^	23.69 ± 0.54^aE^
	S4%	16.88 ± 0.15^bA^	21.09 ± 0.21^bB^	23.18 ± 0.18^bC^	23.28 ± 1.06^bC^	23.31 ± 0.79^aC^
	S5%	16.83 ± 0.19^aA^	19.77 ± 0.29^aB^	22.75 ± 0.14^aC^	23.47 ± 1.20^bD^	24.93 ± 0.16^aE^
b*	S0%	34.67 ± 0.61^eE^	31.33 ± 0.3^aD^	27.21 ± 0.16^bC^	17.93 ± 0.80^aB^	14.58 ± 0.1^aA^
	S1%	33.71 ± 0.18^dE^	31.03 ± 1.26^aD^	26.70 ± 0.15^aC^	18.26 ± 0.26^bB^	15.29 ± 0.45^bA^
	S2%	33.06 ± 0.27^dE^	29.87 ± 1.19^aD^	26.13 ± 1.16^aC^	21.39 ± 1.31^cB^	16.86 ± 0.46^cA^
	S3%	32.14 ± 0.15^cE^	29.54 ± 1.31^aD^	25.68 ± 1.23^aC^	22.86 ± 1.52^dB^	17.58 ± 1.16^cA^
	S4%	31.47 ± 0.34^bE^	29.31 ± 1.36^aD^	25.03 ± 1.16^aC^	24.17 ± 1.49^eB^	18.32 ± 0.26^cA^
	S5%	30.61 ± 0.24^aE^	29.04 ± 1.27^aD^	24.45 ± 1.25^aC^	26.69 ± 1.23^fB^	19.09 ± 0.37^dA^

^1^ Data are presented as mean ± SD (*n* = 3). Mean values within a row with different uppercase superscripts (A–E) and column with different lowercase superscripts (a–f) are significantly different (*p* < .05) from each other. S0%, S1%, S2%, S3%, S4%, and S5% are starch concentrations at 0%, 1%, 2%, 3%, 4%, and 5%, respectively.

#### 3.3.2 Weight loss and firmness

**FIGURE 2:**
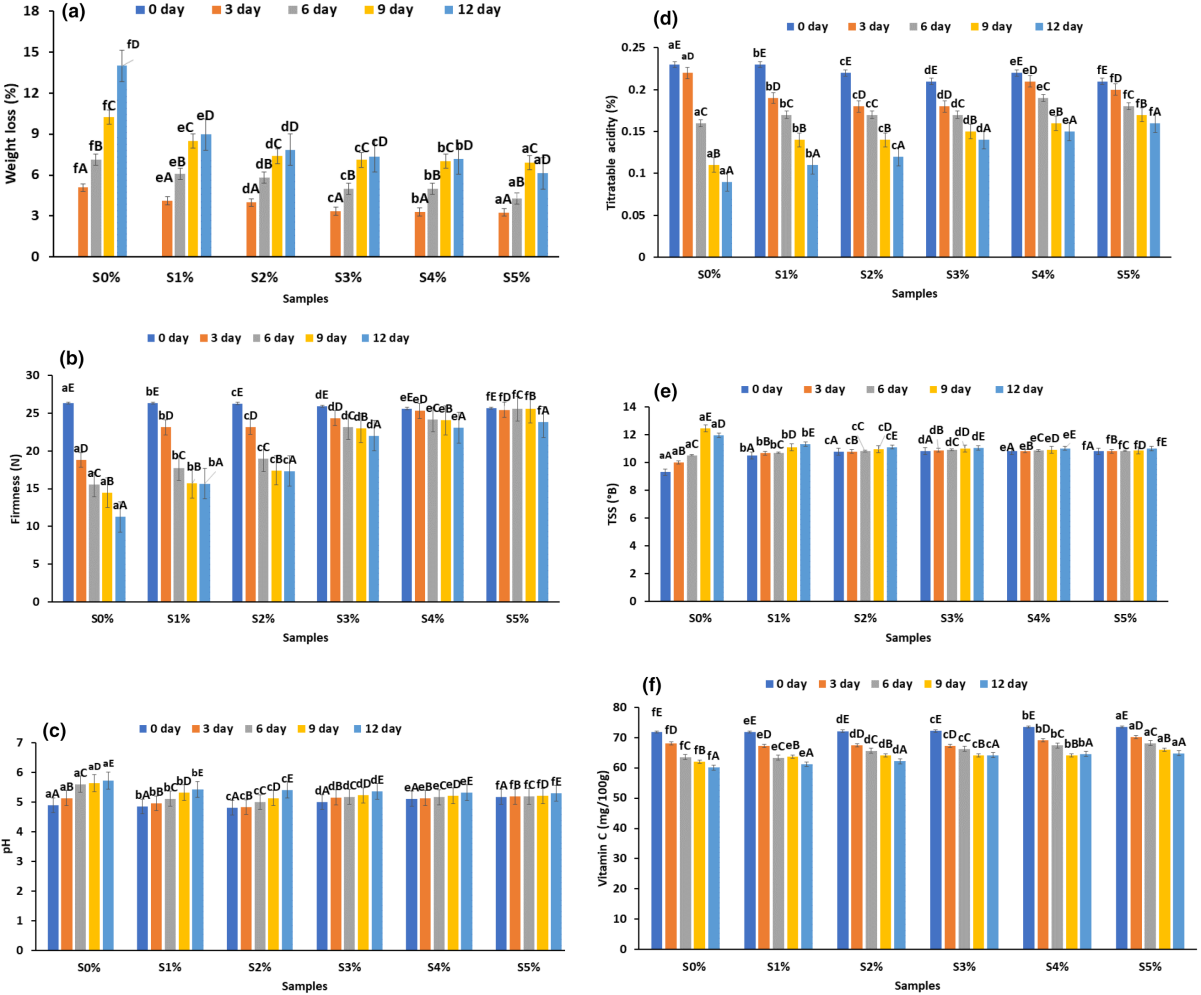
Physicochemical characteristics of edible‐coated cut papaya fruit over 12 days of storage at 4°C. (a) Weight loss, (b) firmness (N), (c) pH, (d) titratable acidity (%), (e) TSS, and (f) vitamin C (mg/100 g). Error bar represents the standard deviation from the mean of three independent replications (*n* = 3), while different lowercase letters (a–f) above each bar represent significantly different (*p* < .05) from each other within the samples and different uppercase superscripts (A–E) above each bar represent significantly different (*p* < .05) from each other within days. TSS, total soluble solids. S0%, S1%, S2%, S3%, S4%, and S5% are starch concentrations at 0%, 1%, 2%, 3%, 4%, and 5%, respectively.

The weight loss of the cut papaya fruits is represented in Figure 2a. A general increasing tendency of weight loss was noted in all treatments during the storage period but on the 12th day of storage, the weight loss percentage was significantly lower (*p* ≤ .05) in the coated samples; S1% (8.96 ± 0.23%), S2% (7.85 ± 0.47%), S3% (7.36 ± 0.86%), S4% (7.18 ± 1.28%), and S5% (6.12 ± 0.59%) as compared to the control samples; S (13.98 ± 0.36%). Moisture loss in the fruits mainly occurs due to the respiration and the movement of carbon dioxide (CO_2_) through the outer layer. The application of starch on fruits acts as a barrier to moisture loss and gas exchange, which can slow down the rate of degradation and diminish the weight loss in coated fruits (Maringgal et al., 2020). The mung bean starch decreases the water vapor permeability, thus improving the barrier against moisture. Similar results have been reported by Francisco et al. (2020) for the guava coated with starch and cellulose‐based films. On the other hand, during storage, the firmness decreased in papaya fruits from all treatments, as shown in Figure 2b. The reduction in firmness of the control sample (11.26 N) was significantly higher (*p* > .05) than that of the coated sample; S1% (15.67 N), S2% (17.34 N), S3% (22.01 N), S4% (23.09 N), and S5% (23.83 N) on the 12th day of storage. The greater loss of firmness in the control sample may due to the water activity and metabolic changes in the cut fruits. The softening of cut fruits can also be due to the pectin hydrolysis. Mung bean starch provides a strong protective coating on the surface of cut fruits, which acts as barrier and prevents moisture loss, which increases the shelf life of cut papaya fruits. Our results align with those of the study of Kathiresan and Lasekan (2019), who applied starch‐based coatings to papaya fruits. Overall, it may be concluded that coating with varying starch concentrations helps in reducing the weight loss and firmness of cut papaya fruit over time compared to the uncoated samples.

#### 3.3.3 pH, titratable acidity, and total soluble solids (TSS)

The pH of both the control and coated samples is shown in Figure 2c. The control samples (5.73 ± 0.18) showed significant increase (*p* > .05) in the pH and a gradual increase was observed in all the coated samples; S1% (5.43 ± 0.76), S2% (5.42 ± 0.34), S3% (5.36 ± 0.23), S4% (5.32 ± 0.37), and S5% (5.31 ± 0.56) on the 12th day of storage. The rise in pH level can be attributed to the fruit ripening and senescence. This leads to a reduction in the fruit's acid content, as acids act as substrates for the respiratory metabolism. As the rate of metabolic respiration increases, the acidity of the fruit tends to decline (Chen et al., 2020). The results align with those of the study of (Kathiresan & Lasekan, 2019) who applied the chickpea starch‐based coating on fresh‐cut papaya.

The titratable acidity of both the samples is presented in Figure 2d, which was decreased during storage. The titratable acidity of the control sample (0.09%) was decreased significantly (*p* < .05) as compared to the coated samples; S1% (0.11 ± 0.23%), S2% (0.12 ± 0.47%), S3% (0.14 ± 0.86%), S4% (0.15 ± 1.28%), and S5% (0.16 ± 0.59%) on the 12th day of storage. The acidity of the fruits is primarily influenced by the rate of metabolism due to respiration and the presence of organic acids. This trend is in line with the findings of the study done by Chettri et al. (2023), suggesting that starch coating could potentially decrease the metabolic changes and slow down the respiration rate in fruits.

The TSS for both the samples are displayed in Figure 2e, which was increased in both the samples during 12 days of storage. Ripening of fruit with time is directly related to increase in TSS content owing to the conversion of polysaccharides into simpler monosaccharide sugars, which is found in all the samples. The initial TSS of control sample was 9.31 ± 0.18°B, which was further increased up to 12.48 ± 0.14°B on the 9th day of storage. However, on the 12th day a decline in TSS was observed in the control samples, which may have been caused by the sugar conversion into acids that is often linked with fruit deterioration. The reduction in sugar content and rise in acidity are observed in the deteriorating fruits. This decrease in sugar content and increase in acidity are common characteristics of decaying fruits (Sharma et al., 2024). On the other hand, the TSS of the starch coated fruits was increased gradually; S1% (11.34 ± 0.12°B), S2% (11.12 ± 0.56°B), S3% (11.06 ± 0.64°B), S4% (11.03 ± 0.35°B), and S5% (11.01 ± 0.64°B), up to the 12th day of storage. The obtained results are similar to those of the study of Kathiresan and Lasekan (2019) on fresh‐cut papaya fruit. Overall, the mung bean starch coating primarily helps in maintaining freshness and extending the shelf‐life of the cut papaya fruit.

#### 3.3.4 Vitamin C analysis

The vitamin C content of both the samples was decreased during 12 days of storage, as presented in Figure 2f. On the 0th day of analysis, the vitamin C content was 71.82 ± 1.45 mg/100 g for the control sample, which was significantly decreased (*p* < .05) on the 12th day of storage (60.10 ± 1.28 mg/100 g). The coated sample showed an insignificant decrease in the vitamin C content during storage; S1% (61.18 ± 1.28 mg/100 g), S2% (62.14 ± 1.28 mg/100 g), S3% (64.18 ± 1.28 mg/100 g), S4% (64.56 ± 1.28 mg/100 g), and S5% (64.82 ± 1.28 mg/100 g). This reduction in the control cut papaya sample may be due to the oxidation and the coated sample was less prone to oxygen, which reduces the respiration rate and consequently preserves the vitamin C content in the coated fruits. A similar result has been found by Ganduri (2020) for vitamin C content of coated banana fruit.

#### 3.3.5 Microbial analysis

Microbial analysis of both papaya samples over a period of 12 days is represented in Table 2. The analysis compared the control papaya fruit with starch‐coated papaya fruits and measurements were taken at regular intervals of 0, 3, 6, 9, and 12 days. Initially, on the 0th day of analysis, no microbial growth was observed in both the samples. However, on the 12th day of analysis, the TPC of the control sample (0.89 ± 0.07 log CFU/g) increased significantly (*p* < .05) as compared to the starch‐coated sample; S1% (0.74 ± 0.10 log CFU/g), S2% (0.61 ± 0.07 log CFU/g), S3% (0.53 ± 0.03 log CFU/g), S4% (0.49 ± 0.08 log CFU/g), and S5% (0.40 ± 0.09 log CFU/g). Similarly yeast and mold count in the control sample (0.47 ± 0.06 log CFU/g) was increased significantly (*p* < .05) in comparison to the coated sample; S1% (0.05 ± 0.47 log CFU/g), S2% (0.45 ± 0.03 log CFU/g), S3% (0.44 ± 0.05 log CFU/g), S4% (0.43 ± 0.08 log CFU/g), and S5% (0.42 ± 0.11 log CFU/g) on the 12th day. Mung bean starch contains a number of antimicrobial compounds, including flavonoids, phenolic acids, and saponins. Some studies have shown that mung bean starch has high antioxidant activity, which may be due to the presence of these phenolic compounds (Shi et al., 2016). Antioxidants from mung bean starch may inhibit microorganism growth by neutralizing free radicals that damage bacteria. Moreover, the formation of mung bean starch films on the surface of fruits creates a physical barrier that restricts the contact and adhesion of microbial agents, thus impeding their growth and proliferation (Bahmid et al., 2023). Furthermore, the addition of glycerol to the coating solution can also contribute to inhibiting microbial growth as it helps to maintain the moisture content, making the environment less favorable for microbial growth. Similar results have been obtained by Chu et al. (2020) for pullulan coating‐based strawberries.

**TABLE 2:** Microbiological count of starch‐coated papaya cut fruits over 12 days of storage at 4°C.^1^

Microbial group	Sample	Time of storage (days)				
		0	3	6	9	12
Total plate count (log CFU/g)	S0%	ND	0.28 ± 0.03^fA^	0.32 ± 0.11^fB^	0.67 ± 0.12^fC^	0.89 ± 0.07^fD^
	S1%	ND	0.25 ± 0.06^eA^	0.31 ± 0.06^eB^	0.53 ± 0.08^eC^	0.74 ± 0.10^eD^
	S2%	ND	0.24 ± 0.09^dA^	0.29 ± 0.09^dB^	0.51 ± 0.03^dC^	0.61 ± 0.07^dD^
	S3%	ND	0.23 ± 0.02^cA^	0.28 ± 0.07^cB^	0.46 ± 0.02^cC^	0.53 ± 0.03^cD^
	S4%	ND	0.20 ± 0.05^bA^	0.27 ± 0.08^bB^	0.39 ± 0.09^bC^	0.49 ± 0.08^bD^
	S5%	ND	0.19 ± 0.04^aA^	0.25 ± 0.05^aB^	0.38 ± 0.04^aC^	0.40 ± 0.09^aD^
Yeast and mold count (log CFU/g)	S0%	ND	0.21 ± 0.01^eA^	0.32 ± 0.08^fB^	0.39 ± 0.03^fC^	0.47 ± 0.06^fD^
	S1%	ND	0.18 ± 0.03^dA^	0.20 ± 0.07^eB^	0.38 ± 0.04^eC^	0.46 ± 0.05^eD^
	S2%	ND	0.17 ± 0.09^cA^	0.20 ± 0.08^dB^	0.37 ± 0.02^dC^	0.45 ± 0.03^dD^
	S3%	ND	0.16 ± 0.04^bA^	0.21 ± 0.06^cB^	0.36 ± 0.05^cC^	0.44 ± 0.05^cD^
	S4%	ND	0.16 ± 0.03^bA^	0.22 ± 0.08^bB^	0.35 ± 0.02^bC^	0.43 ± 0.08^bD^
	S5%	ND	0.15 ± 0.05^aA^	0.23 ± 0.01^aB^	0.34 ± 0.07^aC^	0.42 ± 0.11^aD^

^1^ Data are presented as mean ± SD (*n* = 3). Mean values within a row with different uppercase superscripts (A–D) and column with different lowercase superscripts (a and f) are significantly different (*p* < .05) from each other. CFU, colony‐forming unit; ND, not detected. S0%, S1%, S2%, S3%, S4%, and S5% are starch concentrations at 0%, 1%, 2%, 3%, 4%, and 5%, respectively.

#### 3.3.6 Sensory analysis

**FIGURE 3:**
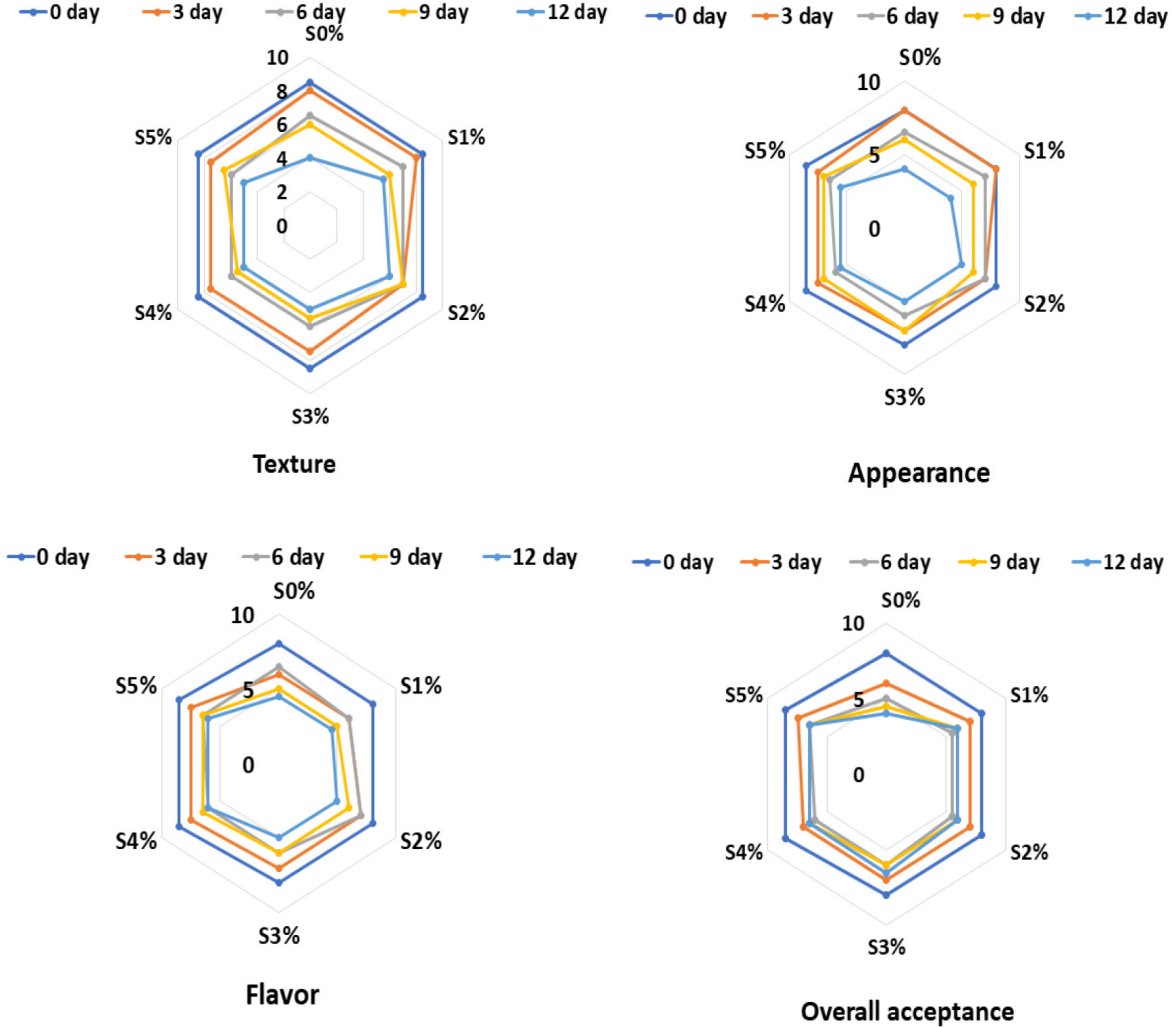
Sensory analysis of edible coating‐treated cut papaya fruits over 12 days of storage (*n* = 35). S0%, S1%, S2%, S3%, S4%, and S5% are starch concentrations at 0%, 1%, 2%, 3%, 4%, and 5%, respectively.

Sensory analysis, including appearance, texture, flavor, and total acceptance of both the samples, is indicated in Figure 3. The least grades emerged out for the control samples as compared to the all‐coated samples based on the 9‐point hedonic scale. The S5% sample showed the highest grades among all other coated samples. The uncoated samples indicated the lowest grades for appearance after 12 days of storage, which may be due to the loss of moisture from the cut papaya fruits. Overall acceptance of coated papaya fruits was 8 on the 0th day, which reduced to 6.5 on the 12th day of analysis.

## 4 CONCLUSION AND FUTURE PERSPECTIVE

The exploration of mung bean starch as a vital component for the development of edible coatings marks a significant advancement in the pursuit of enhancing the shelf‐life of papaya fruits. The observed effects of mung bean starch‐based coatings on the reduction of total soluble solids, maintenance of acidity levels, and the delayed ripening process highlight its potential as a viable solution for the preservation of minimally processed foods. The barriers created by starch against moisture loss, gas exchange, and oxidation serve as effective strategies to retain the freshness and quality of papaya fruits over extended periods. Building on these findings, future perspectives involve exploring deeper into optimizing starch coating formulations, investigating their interactions with various fruit types, and assessing their wider application in the context of sustainable packaging solutions. This research paves the way for innovative approaches to mitigate food wastage and underscores the importance of harnessing nature‐derived materials for the betterment of food preservation techniques.

## AUTHOR CONTRIBUTIONS

**Madhu Sharma:** Conceptualization (equal); data curation (equal); formal analysis (equal); investigation (equal); methodology (equal); writing – original draft (equal). **Aarti Bains:** Conceptualization (equal); investigation (equal); methodology (equal); writing – original draft (equal). **Sanju Bala Dhull:** Resources (equal); software (equal); validation (equal); writing – review and editing (equal). **Prince Chawla:** Conceptualization (equal); data curation (equal); resources (equal); software (equal); supervision (equal); visualization (equal); writing – review and editing (equal). **Gulden Goksen:** Conceptualization (equal); data curation (equal); validation (equal); methodology (equal); supervision (equal); visualization (equal); writing – review and editing (equal). **Nemat Ali:** Funding acquisition (equal); validation (equal); visualization (equal).

## CONFLICT OF INTEREST STATEMENT

The authors declare that they have no known competing financial interests or personal relationships that could have appeared to influence the work reported in this paper.

## Data Availability

The data that support the findings of this study are available from the corresponding author upon reasonable request.
